# The purinergic receptor P2X5 regulates inflammasome activity and hyper-multinucleation of murine osteoclasts

**DOI:** 10.1038/s41598-017-00139-2

**Published:** 2017-03-15

**Authors:** Hyunsoo Kim, Matthew C. Walsh, Noriko Takegahara, Sarah A. Middleton, Hong-In Shin, Junhyong Kim, Yongwon Choi

**Affiliations:** 1 Department of Pathology and Laboratory Medicine, University of Pennsylvania Perelman School of Medicine, Philadelphia, PA 19104 USA; 2 Next generation Optical Immune-imaging, WPI-Immunology Frontier Research Center, Osaka University, Suita, Osaka 565-0871 Japan; 3 Department of Biology, Department of Computer and Information Science, School of Arts and Sciences, Program in Single Cell Biology, University of Pennsylvania, Philadelphia, PA 19104 USA; 4 IHBR, Department of Oral Pathology, School of Dentistry, Kyungpook National University, Daegu, 700412 South Korea

## Abstract

Excessive bone resorption by osteoclasts (OCs) can result in serious clinical outcomes, including bone loss that may weaken skeletal or periodontal strength. Proper bone homeostasis and skeletal strength are maintained by balancing OC function with the bone-forming function of osteoblasts. Unfortunately, current treatments that broadly inhibit OC differentiation or function may also interfere with coupled bone formation. We therefore identified a factor, the purinergic receptor P2X5 that is highly expressed during the OC maturation phase, and which we show here plays no apparent role in early bone development and homeostasis, but which is required for osteoclast-mediated inflammatory bone loss and hyper-multinucleation of OCs. We further demonstrate that P2X5 is required for ATP-mediated inflammasome activation and IL-1β production by OCs, and that P2X5-deficient OC maturation is rescued *in vitro* by addition of exogenous IL-1β. These findings identify a mechanism by which OCs react to inflammatory stimuli, and may identify purinergic signaling as a therapeutic target for bone loss-related inflammatory conditions.

## Introduction

Osteoclasts (OCs) are hematopoietic-derived cells that resorb bone and are required to maintain proper bone homeostasis and skeletal strength by balancing the bone-forming function of osteoblasts (OBs). Under pathogenic inflammatory conditions, excessive bone resorption by OCs can result in serious clinical outcomes, including bone loss that may weaken skeletal or periodontal strength^1, 2^. Such bone resorption is often characterized by increased OCs, OCs that are more efficient at resorbing bone on a per cell basis, and OC “hyper-multinucleation”^2, 3^. Increased OC size and multinucleation is also associated with multiple myeloma and Paget’s disease of bone^4^. However, current treatments for bone loss, bisphosphonates and anti-RANKL antibody (Denosumab)^5^, primarily target early OC commitment and/or later OC viability, and while efficiently anti-resorptive, long-term use may also compromise bone strength due to unintended inhibition of coupled boneformation, which requires positive interplay between OCs (even if non-resorptive) and OBs^6, 7^. Therefore, a better treatment strategy may be to target late-stage OC biological processes (so-called “OC maturation”) in favor of early OC differentiation with the goal of inhibiting OC resorption without preventing secreting activities that contribute to productivebone formation. In this regard, recent clinical trials of the selective cathepsin K inhibitor odanacatib, which specifically targets OCresorption, showed inhibited bone resorption without diminution of either OC generation/survival or coupled bone formation^7, 8^. An emerging idea is that while traditional anti-resorptives will continue to be necessary, they may be prescribed at lower levels in combination with anabolic treatments (such as parathyroid hormone (PTH)) that promote new bone formation; treatments that do not inhibit coupled bone formation are needed to minimize deleterious side effects^7, 8^. Therefore, it will be critical to better understand the regulatory factors and mechanisms that control OC maturation.

Under homeostatic conditions, a hallmark of osteoclast maturation is multinucleation – cellular fusion of multiple immature OCs, typically between three and five^9^, into a single multinucleated cellular entity. However, under some pathological inflammatory conditions, hyper-multinucleation can lead to OCs that contain up to 100 nuclei^10, 11^. This aspect of OC biology bears some similarity to the foreign body-induced inflammatory response, in which macrophage-derived multinucleated foreign body giant cells (FBGCs) are generated^12^ and macrophages fuse to become multinucleated giant cells (MGCs) during granuloma formation^13^. We reasoned that inflammatory macrophages and hyper-multinucleated osteoclasts, while differing in gene expression profiles related to lineage commitment and differentiation, likely share molecular programs that govern hyper-multinucleation and that identification of genes that control OC hyper-multinucleation could be useful in distinguishing the signaling pathways that promote normal osteoclast homeostasis versus osteoclast-mediated inflammatory bone loss. By comparative gene expression profiling, we identified the gene *P2rx5*, which codes the protein P2X5, a purinergic receptor family member, as a specific regulator of OC maturation and hyper-multinucleation, but not of early OC differentiation. Purinergic P2X family proteins are plasma membrane receptors expressed by a wide range of mammalian cells including neurons and glial cells in the central (CNS) and peripheral (PNS) nervous systems, muscle cells, epithelial cells, endothelial cells, endocrine cells, bone cells, and immune cells^14, 15^. These receptors are sensors for extracellular adenosine triphosphate (ATP), an important signaling molecule, and upon ATP binding, mediate calcium flux, induce large pore formation, and form signaling complexes with interacting proteins and membrane lipids. It has been observed for decades that ATP signaling exerts modulating effects on bone homeostasis and expression of various P2X members has been detected in both OCs and OBs^16, 17^. Purinergic receptor signaling has even been implicated in cell fusion and multinucleation processes of inflammatory macrophages^18, 19^. Importantly, purinergic receptor signaling is also linked to inflammatory responses through activation of the inflammasome and release of mature forms of IL-1β and IL-18^20, 21^, which are both relevant to bone homeostasis^2^. We report here that P2X5 expression by murine OCs is required for OC hyper-multinucleation and for ATP-dependent inflammasome activation and secretion of multinucleation-enhancing IL-1β by OCs. We further show that P2X5-deficient mice exhibit normal early bone development and homeostasis, but diminished OC responsiveness to inflammatory conditions *in vivo*. Interestingly, a single nucleotide polymorphism (SNP) found in the 3’ splice site of human *P2rx5* exon 10 in the majority of the population results in a truncated form of the P2X5 protein that lacks parts of both the ATP-binding domain and the second transmembrane domain^22, 23^. Therefore, while it remains unclear, from a population perspective, to what extent P2X5 regulates human physiology, the results we report here may still point to purinergic signaling, broadly, as a promising target for selectively inhibiting inflammatory bone loss.

## Results

### Expression of *P2rx5* is associated with cellular multinucleation

**Figure 1:**
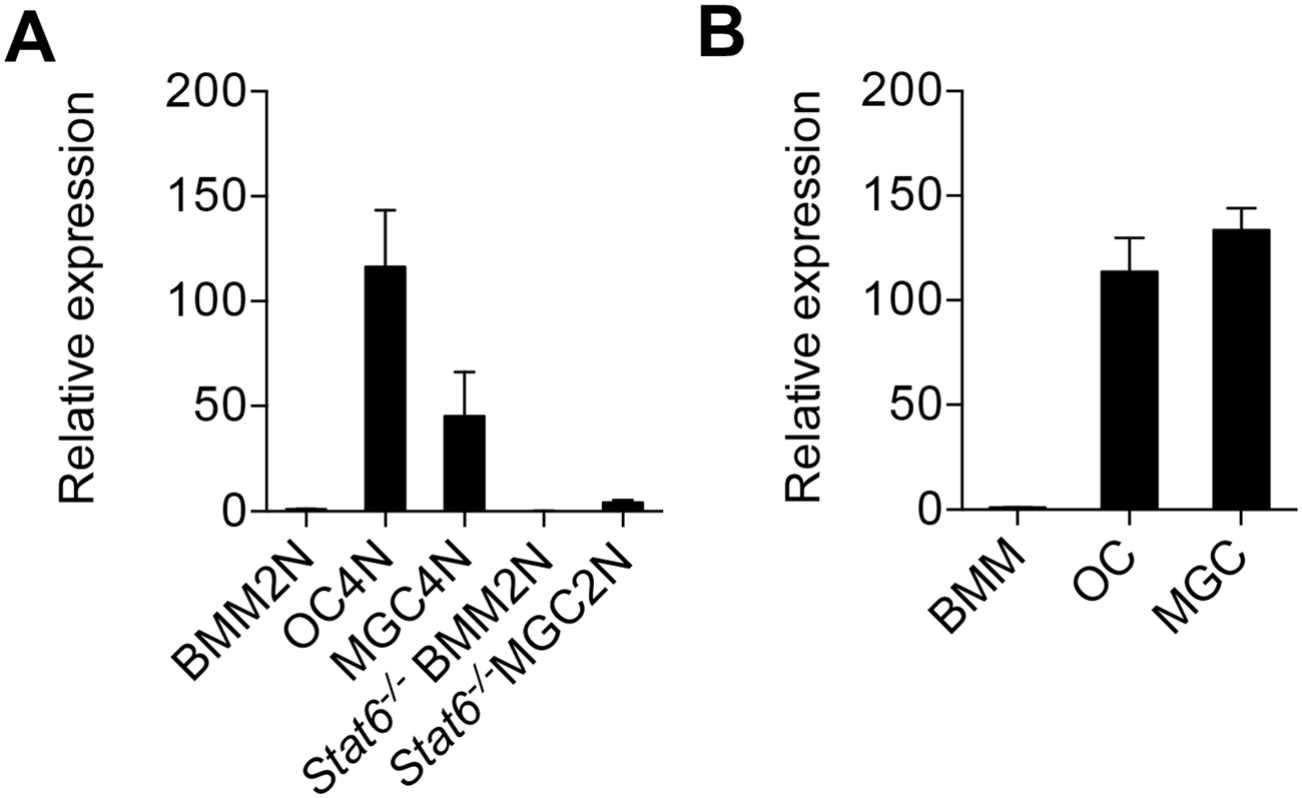
Expression of *P2rx5* is associated with cellular multinucleation. (**A**) Expression of *P2rx5* in pre-commitment mono-nuclear cells and multinucleation-committed bi-nucleated cells. Relative expression of *P2rx5* compared to bi-nuclear BMMs (BMM2N). Multinucleation-committed bi-nucleated cells were sorted from RANKL + M-CSF-induced OCs or from IL-3 + IL-4-induced MGCs, and pre-commitment mono-nuclear cells were sorted from M-CSF-cultured BMMs, or from IL-3 + IL-4-induced *STAT6* ^−/−^ MGCs. Total RNAs were isolated, and all transcripts were sequenced by RNA sequencing. Results of the expression of *P2rx5* are shown as mean ± SD of three independent experiments. (**B**) qPCR validation of *P2rx5* expression in BMMs, OCs and MGCs. Relative expression of *P2rx5* compared to BMMs is shown. Data were normalized to 18s rRNA and are shown as mean ± SD.

In order to identify novel gene targets critical to OC maturation, we used multinucleation as a functional proxy, and looked for genes expressed in common with multi-nucleated giant cells (MGCs). Specifically, monocytes were cultured with either MCSF + RANKL to differentiate toward the OC lineage, or IL-3 + IL-4 to generate MGCs, which also undergo cell fusion. Gene expression was assessed in precommitment mono-nuclear macrophages (BMM2N) versus bi-nucleated OCs (OC4N). Genes associated specifically with early OC lineage commitment were filtered out by identifying overlapping gene expression patterns in bi-nucleated MGCs (MGC4N). Because MGC fusion is dependent on STAT6-mediated IL-4 signaling^24^, we also cultured *STAT6* ^−/−^ monocytes with IL-3 + IL-4 to filter out potentially cell fusion-independent MGC-associated genes. Expression of *P2rx5* was found significantly increased in both sorted OC4N and MGC4N populations compared to baseline BMM2N expression, but increased expression was not observed in 2N populations sorted from *STAT6* ^−/−^ BMM or MGC cultures (Fig. 1A). We confirmed that *P2rx5* message was significantly increased in total OC and MGC versus BMM cultures (Fig. 1B). Therefore, *P2rx5* was selected as a potential regulator of OC maturation.

### Function and expression of purinergic receptors during osteoclast maturation

**Figure 2:**
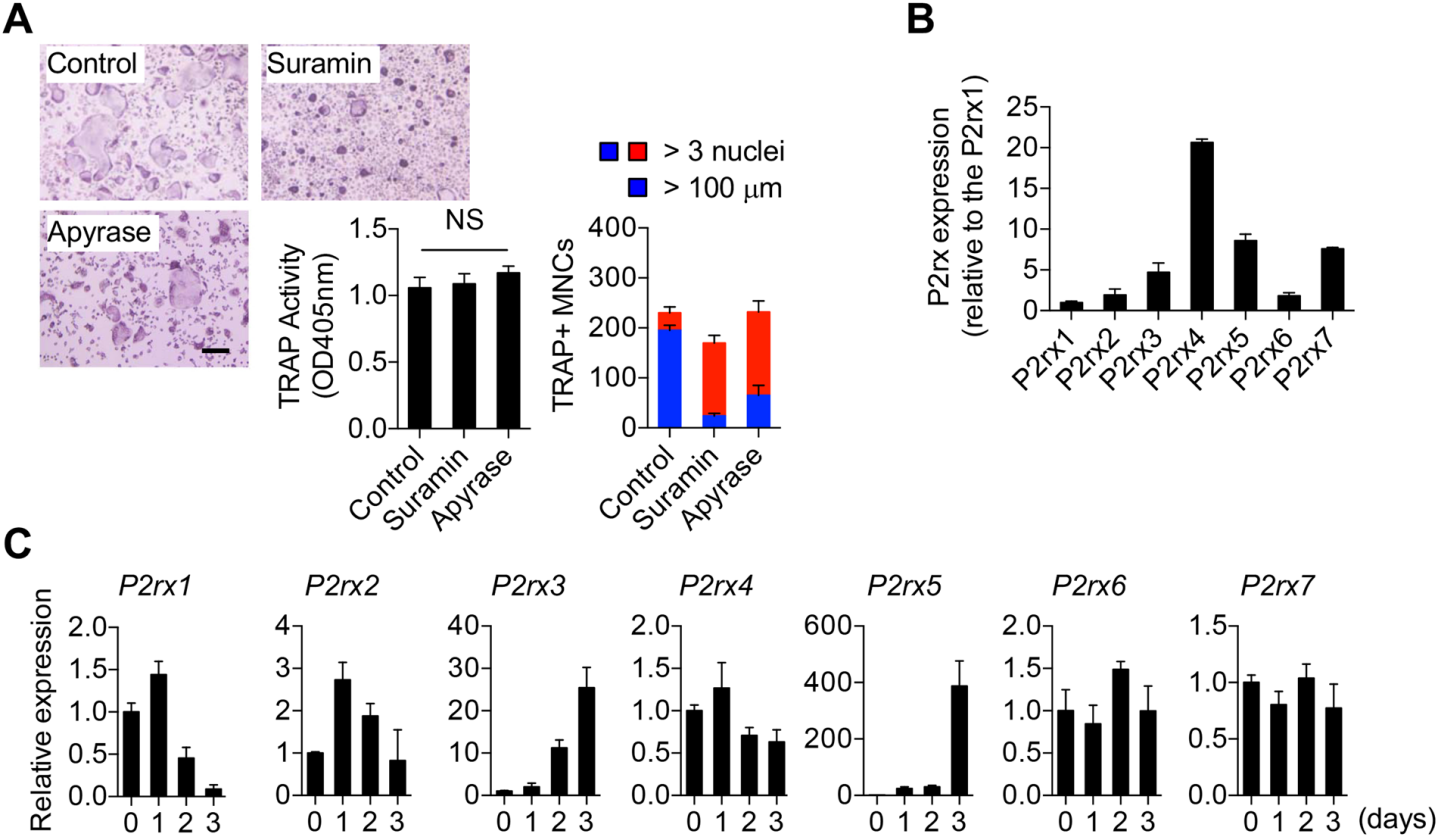
Function and expression of purinergic receptors during osteoclast maturation. (**A**) Effect of the purinergic receptor antagonist suramin on OC differentiation. BMMs were cultured for 3 days +/− suramin (20 μM) in the presence of M-CSF (30 ng/ml) and RANKL (150 ng/ml). Cells were then stained for TRAP and observed by microscopic examination. OCs with more than 3 nuclei and 100 μm in size were counted. Scale bar, 100 μm. NS, not significant. **P < 0.01, ***P < 0.001. Data are shown as means ± SD. (**B**) Comparative expression of *P2rx* subfamily members in mature OCs as determined by qPCR analysis of mRNA. (**C**) Relative induction of *P2rx* subfamily members during OC differentiation/maturation culture as determined by qPCR analysis of mRNA. Data are shown as means ± SD.

To determine whether purinergic receptor signaling may be selectively required for OC maturation versus early differentiation, we treated OC cultures with the P2X antagonist suramin or the ATP diphosphatase apyrase^25^, and found that after 3 days, while there was no significant difference in total tartrate-resistant acid phosphatase (TRAP) activity, an indicator of OC lineage commitment, both the numbers of multinucleated (>3 nuclei per cell) and large, hyper-multinucleated (>100 μm) OCs per well were significantly reduced with suramin or apyrase treatment (Fig. 2A). It should be noted here that for reasons that remain unclear, hyper-multinucleation has long been observed during *in vitro* OC cultures, even without overt addition of inflammatory stimuli^26, 27^, and in this study we have co-opted this trait to better focus on mechanisms that particularly affect OC hyper-multinucleation. We performed qPCR analysis of P2X family members in mature OCs to show relative expression of each gene and found *P2rx4* to be the most highly expressed, followed by *P2rx5* and *P2rx7* (Fig. 2B). We then performed temporal qPCR expression analysis and showed that *P2rx5* exhibits the most dramatic positive difference in expression among the various P2X family members (significantly more even than *P2rx3*, which exhibits a similar expression pattern) during the transition from early (day 0; bone marrow macrophages (BMMs)) to late (day 3 post-RANKL addition) OC cultures (Fig. 2C). These data suggest that P2X5 may be a critical regulator of OC maturation.

### Inhibition of *P2X5* suppresses *in vitro* OC maturation

**Figure 3:**
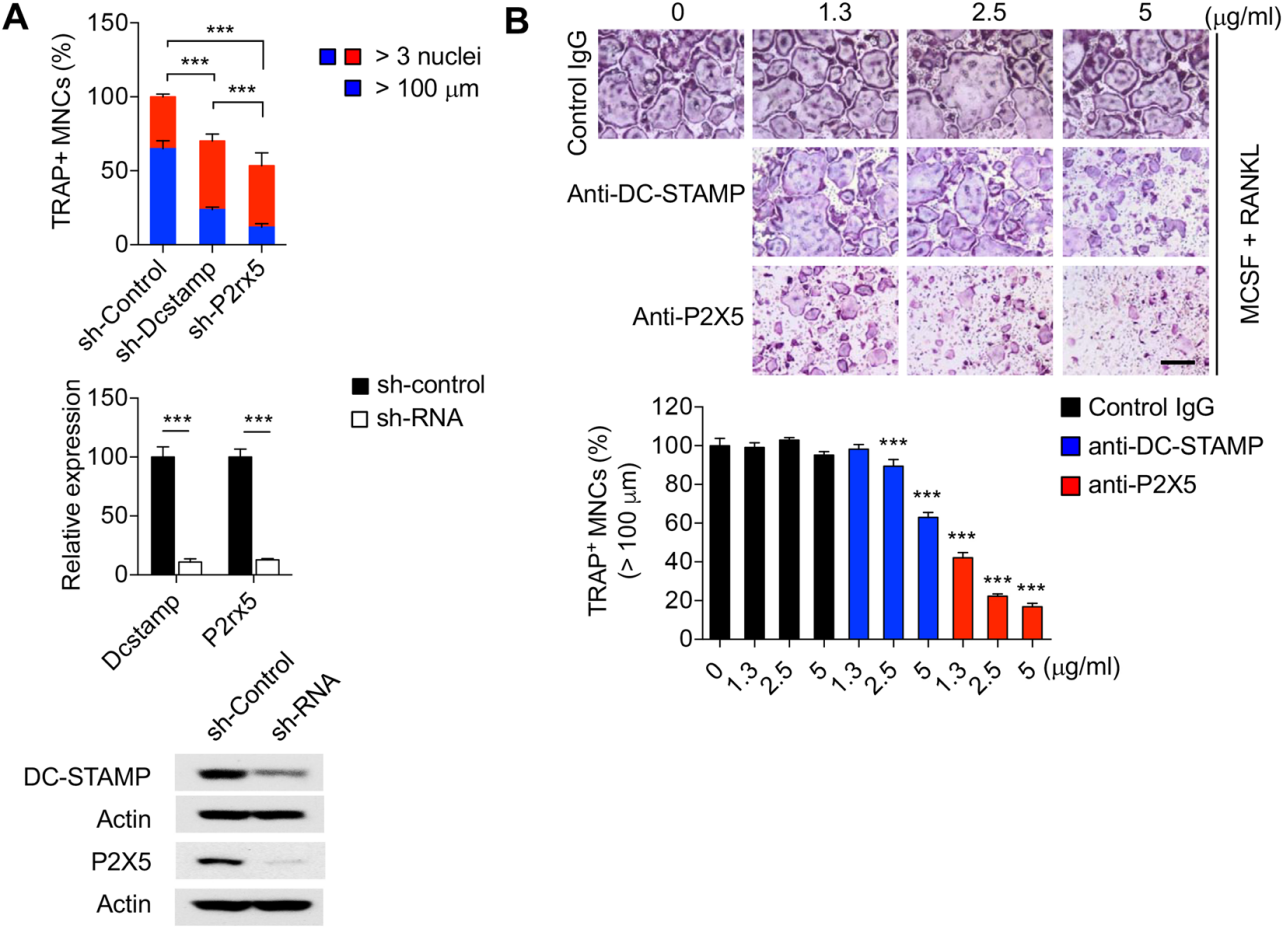
Inhibition of P2X5 suppresses *in vitro* OC maturation. (**A**) TRAP^+^ MNCs in mature (3 days post-addition of RANKL) OC cultures retrovirally-transduced with control shRNA, or shRNAs against *Dcstamp* or *P2rx5*, upper panel. Relative (to control shRNA-tranduced cells) expression of *Dcstamp* or *P2rx5* in mature OC cultures retrovirally-transduced with control shRNA, or shRNAs against *Dcstamp* or *P2rx5*, as determined by qPCR, middle panel. Western blot analysis of expression of DC-STAMP or P2X5 in mature OC cultures retrovirally-transduced with control shRNA, or shRNAs against *Dcstamp* or *P2rx5*, lower panel. (**B**) OC cultures treated with increasing concentrations of control IgG, inhibitory anti-DC-STAMP, or inhibitory anti-P2X5 polyclonal antibodies. The upper panel shows the effects on day 3 OC cultures and the lower panel shows quantitation of decreases in TRAP^+^ MNCs relative to control IgG treatment. Scale bar represents 100 μm. ***P < 0.001. Data are shown as means ± SD.

To specifically investigate the role of P2X5 in OC maturation and multinucleation, we retrovirally transduced BMMs with a construct encoding shRNA for *P2rx5*. As controls we employed an empty vector or shRNA for *Dcstamp*, which encodes DC-STAMP, a critical OC cell fusion regulator^28^. We found that the frequency of large (>100 μm) TRAP^+^ MNCs in OC cultures was significantly decreased with either *P2rx5* or *Dcstamp* shRNA compared to control vector, and that *P2rx5* shRNA was even more inhibitory than *Dcstamp* shRNA (Fig. 3A). To determine the effects of targeting P2X5 protein directly during OC maturation, OC cultures were treated with increasing doses of polyclonal anti-P2X5 antibody. Control IgG and anti-DC-STAMP antibodies were used as negative and positive control inhibitors, respectively. Like, shRNA expression, we found that anti-P2X5 antibody significantly reduced the percentage of TRAP^+^ MNCs relative to DC-STAMP inhibition (Fig. 3B), again suggesting that P2X5 may be a suitable target for inhibiting OC maturation, and one that may be targeted directly via the extracellular domain.

### *P2rx5* deficiency suppresses *in vitro* OC maturation

**Figure 4:**
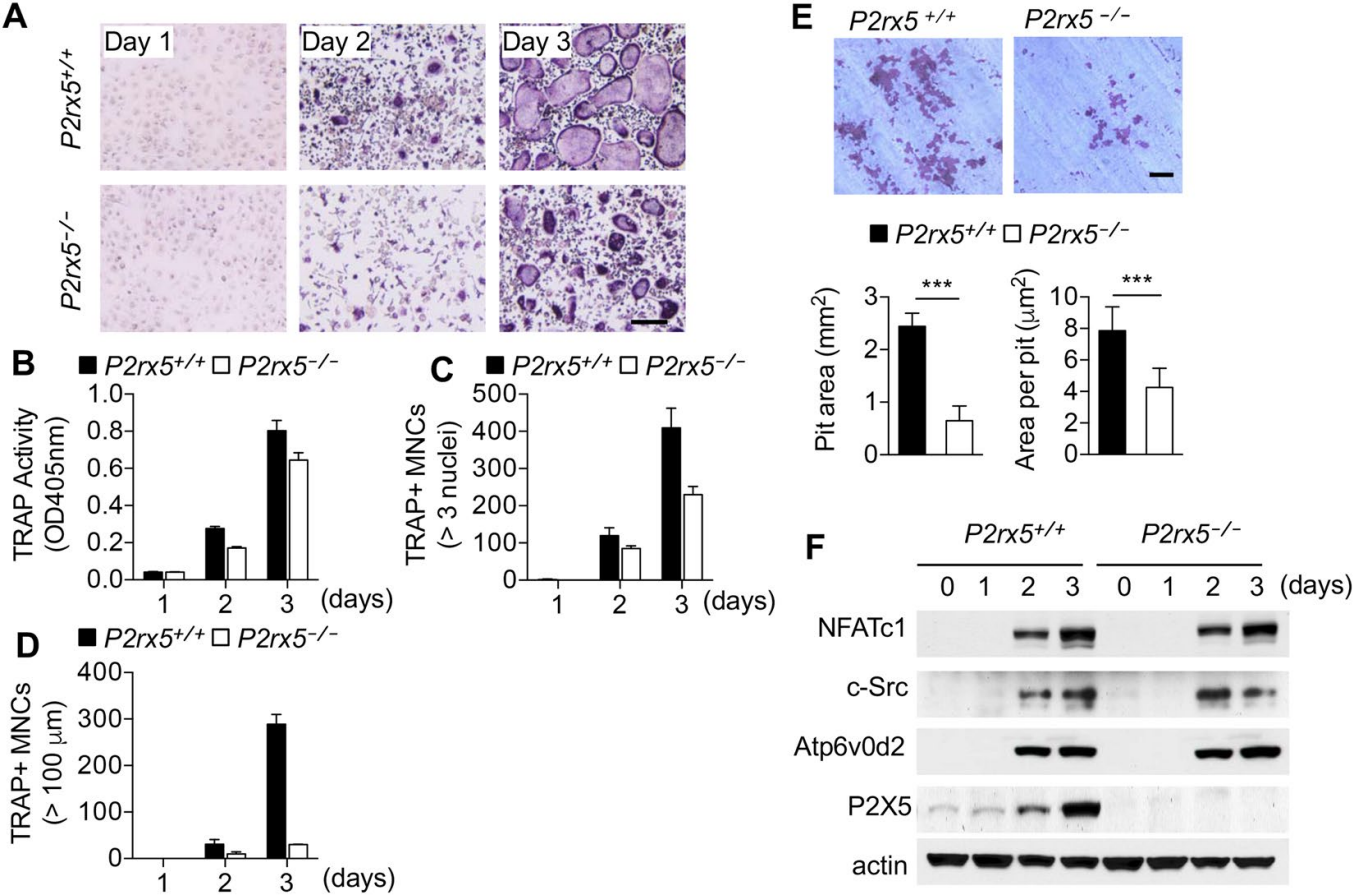
*P2rx5* deficiency suppresses *in vitro* OC maturation. (**A–D**) *P2rx5* ^+/+^ and *P2rx5* ^−/−^ BMMs were cultured for the indicated times in the presence of M-CSF and RANKL. (**A**) Bright-field images of OCs stained for TRAP and (**B**) quantitation of TRAP activity in OCs measured at OD405nm. TRAP^+^ MNCs were enumerated both by (**C**) the presence of more than 3 nuclei and (**D**) cell size larger than 100 μm in diameter. Scale bar represents 100 μm. (**E**) Bone resorption activity of *P2rx5* ^+/+^ and *P2rx5* ^−/−^ during OC differentiation. BMMs were cultured on dentine slices for 3 days with M-CSF and RANKL to induce OC differentiation, then OCs were further incubated for 2 additional days, and pit areas analyzed. Resorption area (left plot) and resorption area per OC (right plot). Scale bar represents 200 μm. ***P < 0.001. Data are shown as means ± SD. (**F**) Western blot analysis of NFATc1, c-Src, Atp6v0d2 and P2X5 expression during osteoclast differentiation. Lysates were made at indicated time points from *P2rx5* ^+/+^ and *P2rx5* ^−/−^ OC cultures. Probing against actin was performed for normalization.

To determine the specific cell-intrinsic role of *P2rx5* during OC differentiation and maturation, we generated *P2rx5* ^−/−^ mice, and cultured *P2rx5* ^+/+^ and *P2rx5* ^−/−^ BMMs in the presence of M-CSF and RANKL for up to three days (Fig. 4A). While minor reduction of total TRAP activity was seen in *P2rx5* ^−/−^ compared to *P2rx5* ^+/+^ cultures by day three (Fig. 4B), the reduction in numbers of multinucleated (>3 nuclei) (Fig. 4C), and particularly in hyper-multinucleated (>100 μm) (Fig. 4D), OCs per well were far more dramatic. Also, use of anti-P2X5 blocking antibody showed reduced activity in *P2rx5* ^−/−^ compared to *P2rx5* ^+/+^ cultures (Fig. S1). Importantly, the cumulative functional effects of P2X5 deficiency-related differences were revealed by resorption pit analyses, showing reduced pit numbers and overall resorption area (Fig. 4E). At the same time, temporal analyses of protein expression in *P2rx5* ^+/+^ and *P2rx5* ^−/−^ lysates prepared from OC cultures showed that while P2X5 expression peaks in *P2rx5* ^+/+^ cells at day three of RANKL treatment, critical OC differentiation and maturation factors NFATc1^29^, c-Src^30^, and Atp6v0d2^31^ (Fig. 4F) are not affected by P2X5 deficiency, suggesting that P2X5 may function in a manner independent of regulating NFATc1, c-Src, or Atp6v0d2 expression. Additional comparative qPCR analyses of message from *P2rx5* ^+/+^ and *P2rx5* ^−/−^ OC cultures showed that while expression for the OC differentiation and maturation markers acid phosphatase 5, tartrate-resistant (ACP5), nuclear factor of activated T cells, cytoplasmic 1 (NFATc1), c-fos, Atp6v0d2, DC-STAMP showed no significant differences, some others, including OSCAR, cathepsin K (Ctsk), carbonic anhydrase 2 (Car2) and Integrin β3 (Itgb3) did show reduced expression in *P2rx5* ^−/−^ OC samples, particularly at later time points (Fig. S2).

### Normal bone development and homeostasis in *P2rx5*^−/−^ mice

**Figure 5:**
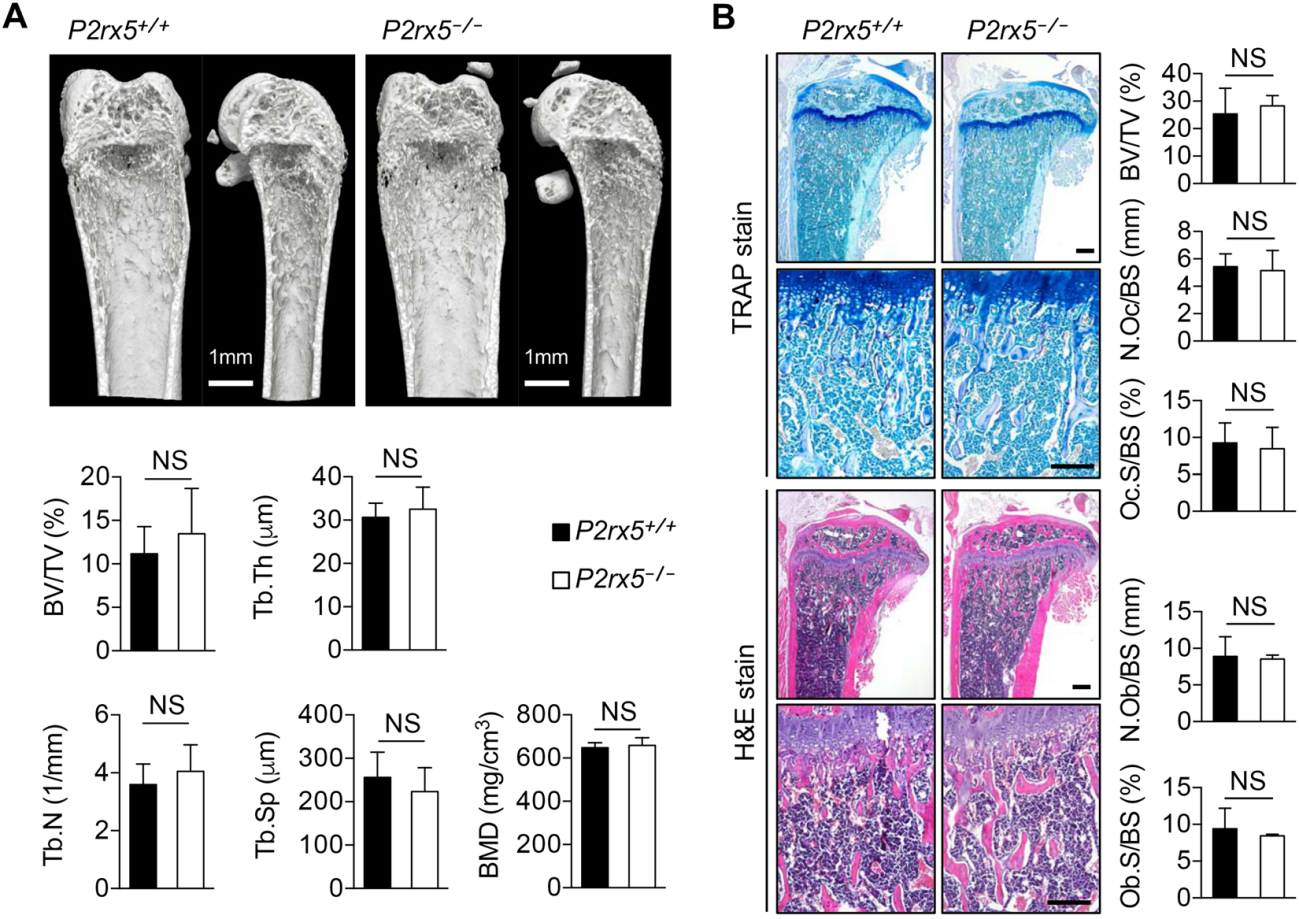
Normal bone development and homeostasis in *P2rx5* ^−/−^ mice. (**A**) Representative 3D reconstructions of trabecular bone. The tibias of 8-week-old male *P2rx5* ^+/+^ and *P2rx5* ^−/−^ littermate mice were analyzed by μCT scanning. Calculations of bone volume per tissue volume (BV/TV), trabecular thickness (Tb.Th), trabecular number (Tb.N), trabecular space (Tb.Sp) and bone mineral density (BMD) are shown. Scale bar, 1mm. NS, not significant. Data are means ± SD. (**B**) Histomorphometric analyses, representative TRAP staining (upper panel) and H&E staining (lower panel). Also shown are BV/TV, osteoclast number per bone surface (N.Oc/BS), OC surface per bone surface (Oc.S/BS), osteoblast number per bone surface (N.Ob/BS) and osteoblast surface per bone surface (Ob.S/BS). Scale bar represents 100 μm. NS, not significant. Data are means ± SD.

We examined the effects of P2X5 deficiency on bone development and homeostasis. Using gender-matched *P2rx5* ^+/+^ and *P2rx5* ^−/−^ littermate mice, representative 3D reconstructions of trabecular bone were performed. Tibia were analyzed by μCT scanning in order to calculate bone volume per tissue volume (BV/TV), trabecular thickness (Tb.Th), trabecular number (Tb.N), trabecular space (Tb.Sp), and bone mineral density (BMD). No significant differences were detected between *P2rx5* ^+/+^ and *P2rx5* ^−/−^ for any measured parameter (Fig. 5A). Further, by histomorphometric analyses, TRAP and H&E stainings were performed and calculations made of BV/TV, osteoclast number per bone surface (N.Oc/BS), osteoclast surface per bone surface (Oc.S/BS), osteoblast number per bone surface (N.Ob/BS) and osteoblast surface per bone surface (Ob.S/BS). Again, no significant differences were detected (Fig. 5B). These data suggest that P2X5 is not required for normal, healthy, bone development or homeostasis.

### Decreased inflammatory bone loss in *P2rx5*^−/−^ mice

**Figure 6:**
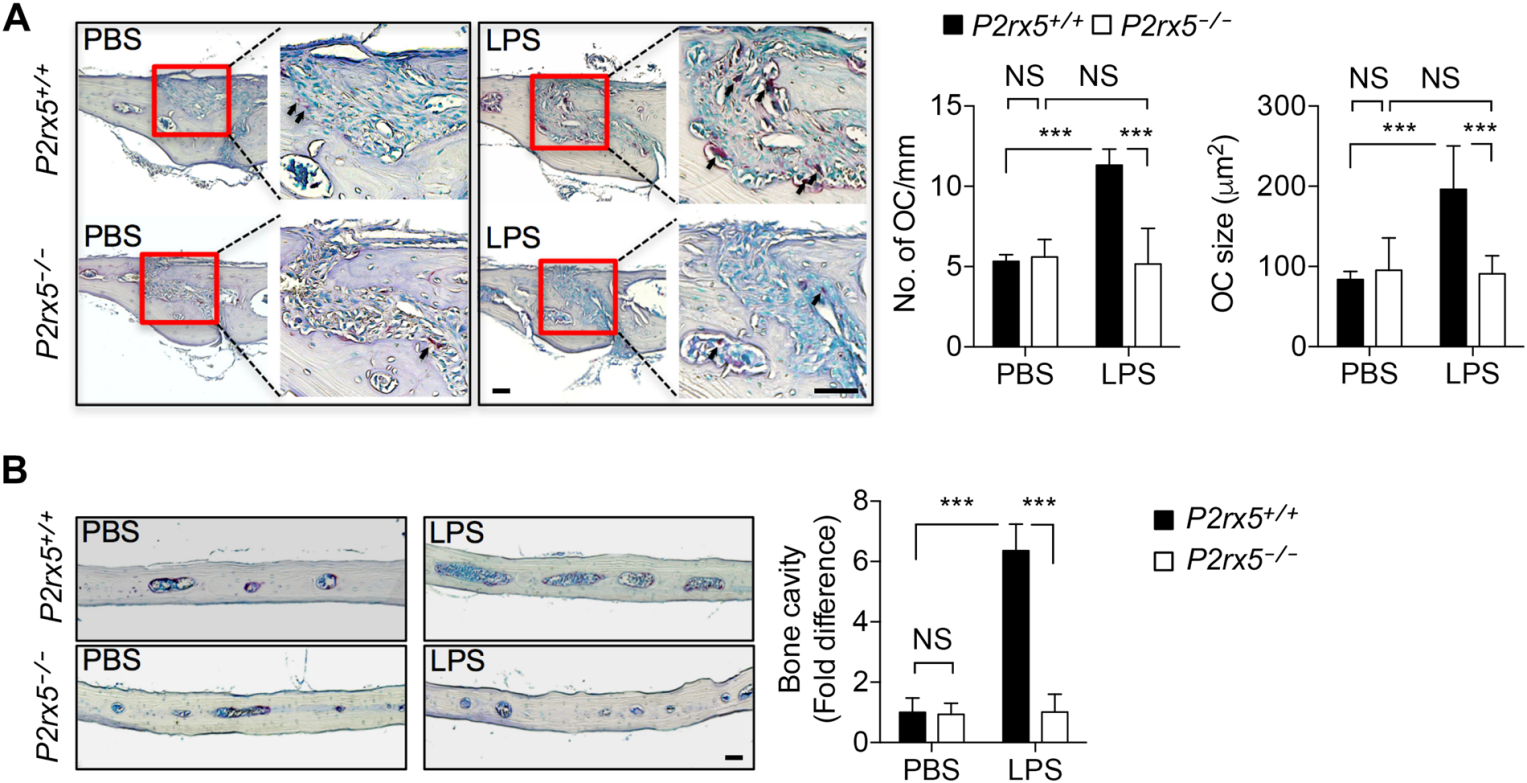
Decreased inflammatory bone loss in *P2rx5* ^−/−^ mice. (**A**) TRAP staining of histological sections of the sagittal suture, with arrows indicating TRAP^+^ OCs (left panel), and quantitation of the number and mean size (shown as mean OC area) of OCs (right panel). ***P < 0.001. Data are means ± SD. (**B**) Resorption cavities in the parietal calvarium tissue area were measured for PBS- and LPS-treated *P2rx5* ^+/+^ and *P2rx5* ^−/−^ mice, and then normalized to the measurement for PBS-treated *P2rx5* ^+/+^ samples to determine fold differences. Scale bar represents 100 μm. ***P < 0.001. Data are means ± SD.

Having established that normal bone homeostasis is intact in the absence of P2X5 expression, we sought to investigate whether our initial hypothesis that hyper-multinucleation-associated genes, such as *P2rx5*, would be more specific for pathologic inflammatory conditions. Therefore, we employed a model of inflammatory bone loss in which LPS is injected above the calvarial bone, resulting in increased OC numbers and localized bone resorption^32, 33^. Gender-matched *P2rx5* ^+/+^ and *P2rx5* ^−/−^ littermate mice were injected with LPS or PBS, followed by TRAP staining of histological sections of the sagittal suture and enumeration of localized osteoclasts, which were significantly increased in *P2rx5* ^+/+^ mice in response to LPS, but not in *P2rx5* ^−/−^ mice (Fig. 6A). Importantly, the mean size of localized OCs (which correlates with the degree of multinucleation) was only found to have increased in LPS-treated (compared to PBS-treated) *P2rx5* ^+/+^, but not *P2rx5* ^−/−^ mice (Fig. 6A). Consistent with these findings, while LPS treatment significantly increased the fold difference of bone cavity of parietal calvarium in *P2rx5* ^+/+^ mice, *P2rx5* ^−/−^ mice treated with LPS exhibited bone cavity profiles similar to PBS-treated controls (Fig. 6B). These data suggest that OC-mediated bone homeostasis may exhibit sensitivity to P2X5 function specifically under inflammatory conditions.

### *P2X5*-mediated inflammasome activation and IL-1β production enhance OC maturation

**Figure 7:**
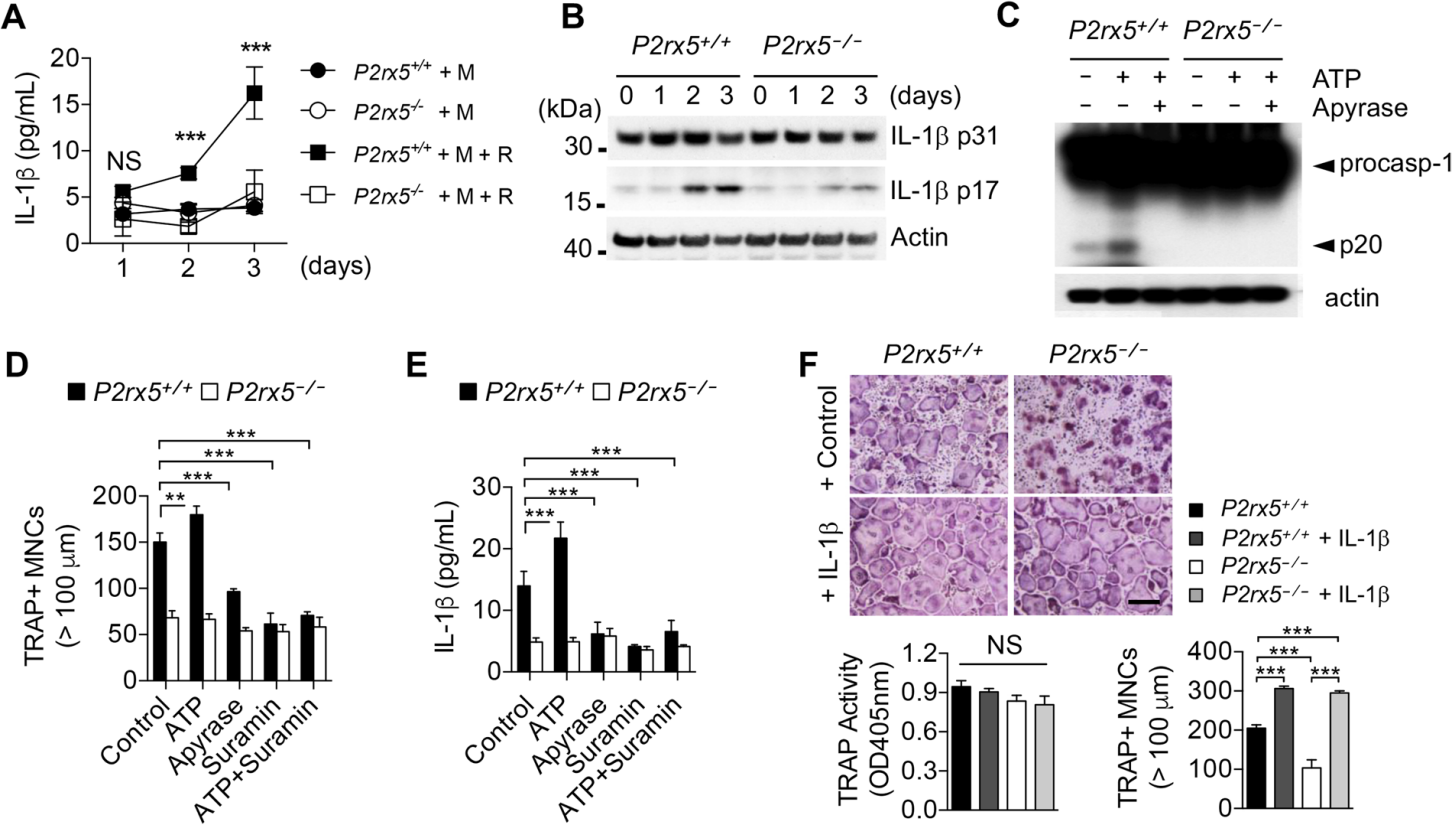
P2X5-mediated inflammasome activation and IL-1β production enhance OC maturation. (**A**) Production of IL-1β during OC differentiation. Levels of IL-1β in culture supernatants were determined by ELISA. ***P < 0.001. NS, not significant. Data are means ± SD. **(B)** Pro-IL-1β and mature-IL-1β production during OC differentiation. BMMs were cultured for the indicated time with M-CSF and RANKL. Western blot analysis was performed to assess the expression of Pro-IL-1β and mature-IL-1β during OC differentiation. At days 0, 1, 2 and 3, whole cell extracts were subjected to western blot analysis with the indicated antibodies. The results are representative of at least two independent sets of similar experiments. (**C**) Activation of caspase-1 at day 3. BMMs were cultured for 3 days in the presence of M-CSF and RANKL. Cultured cells treated with apyrase (10 units/ml) 1 hr before +/− addition of extracellular ATP (100 μM) for 1 hr then subjected to western blot analysis with the anti-caspase-1 (p20) and anti-actin. (**D** and **E**) BMMs were cultured for 2 days in the presence of M-CSF and RANKL, then cultured for 1 day in the presence of ATP, apyrase and/or suramin (20 μM). **P < 0.01, ***P < 0.001. NS, not significant. Data are shown as means ± SD. (**D**) Enumeration of >100 μm TRAP^+^ MNCs per culture well. (**E**) Levels of IL-1β in culture supernatants were determined by ELISA. ***P < 0.001. Data are means ± SD. (**F**) BMMs were cultured for 2 days in the presence of M-CSF and RANKL, then cultured for 1 day with or without IL-1β. Cells were fixed and stained for TRAP activity (*lower left panel*), and TRAP^+^ MNCs were enumerated (*lower right panel*). Scale bar represents 100 μm.

Having shown both decreased hyper-multinucleation of *P2rx5* ^−/−^ OCs *ex vivo*, and mitigation of inflammatory bone loss in *P2rx5* ^−/−^ mice, we sought to identify OC-intrinsic mechanisms of inflammation potentially regulated by P2X5. Because of the known role of purinergic signaling in inflammasome activation, we assayed production of IL-1β in OC cultures, and observed a significant defect in *P2rx5* ^−/−^ compared to *P2rx5* ^+/+^ samples, particularly at later time points (Fig. 7A). At the same time, no differences in expression of the immature form of IL-1β were observed (Fig. 7B), suggesting that P2X5 does not regulate expression of the IL-1β gene. In addition to IL-1β maturation, inflammasome activation is characterized by cleavage of caspase-1. To formally test P2X5 dependence of inflammasome activation, we treated day two post-RANKL-treated OC cultures with vehicle, the purinergic receptor ligand ATP, or the ATP inhibitor apyrase, and then assayed caspase-1 cleavage by western blot. We found that apyrase-sensitive ATP-dependent cleavage of caspase-1 to p20 was observed in *P2rx5* ^+/+^ but not *P2rx5* ^−/−^ samples (Fig. 7C). Notably, some caspase-1 cleavage was observed even in the absence of addition of supplementary ATP. It has previously been shown that cellular ATP is modulated during OC differentiation^34–36^. Though we have found that P2X5 deficiency does not affect intracellular or extracellular levels of ATP in OC cultures (Fig. S3), to further investigate the effect of ATP-mediated signaling on downstream aspects of OC biology and whether supplementary ATP can rescue OC defects related to P2X5 deficiency, OC cultures were treated with combinations of ATP, apyrase and/or the P2X inhibitor suramin. While supplementary ATP had little effect on either *P2rx5* ^+/+^ or *P2rx5* ^−/−^ cultures, both apyrase and suramin exerted a similar, and *not synergistic*, inhibitory effect on *P2rx5* ^+/+^ cultures with respect to both large (>100 μm) TRAP^+^ MNC per well (Fig. 7D), and IL-1β (Fig. 7E) production, suggesting each inhibitor may be targeting distinct molecular targets within the same pathway. Similarly, while *P2rx5* ^−/−^ cultures had significant baseline defects, neither suramin nor apyrase alone or together exerted any significant additional inhibitory effects (Fig. 7D and E). Finally, to determine whether enhancing the levels of IL-1β could rescue the maturation and multinucleation defects observed in *P2rx5* ^−/−^ OC cultures, we performed a standard three-day *in vitro* OC cultures with *P2rx5* ^+/+^ and *P2rx5* ^−/−^ BMMs treated with or without supplementary recombinant IL-1β for the final day of culture (Fig. 7F). While overall TRAP activity was unaffected by IL-1β, we found that IL-1β exerted significantly increased enhancement of per well large (>100 μm) TRAP^+^ MNC numbers (over IL-1β-untreated cultures) in *P2rx5* ^−/−^ compared to *P2rx5* ^+/+^ OC cultures. Together, these data suggest that OC-expressed P2X5 is necessary for optimal inflammasome activation and IL-1β production, and that P2X5-dependent OC maturation defects may be rescued by IL-1β.

## Discussion

We have identified the purinergic receptor P2X5 as a critical molecular actor in regulating osteoclast maturation and hyper-multinucleation. However, unlike factors previously identified as important to osteoclast fusion per se, for which genetic deficiencies result in clear defects in skeletal development and homeostasis, such as DC-STAMP^28^ and Fra-2^37^, we found that P2X5-mediated control of OC multinucleation may be restricted to inflammatory conditions, such as those often associated with pathologic bone loss. Importantly, P2X5 seems to play no apparent role in early bone development or homeostasis, and therefore may serve as a suitable therapeutic target for selectively inhibiting pathologic OC activity. We have also demonstrated that hyper-multinucleation as a cell biological process may serve as a proxy for identifying late-stage OC maturation-associated factors as distinct from early OC differentiation factors. Hyper-multinucleation has long been observed during *in vitro* OC cultures even without overt addition of inflammatory stimuli^26, 27^ for reasons that are unclear. It is possible that OCs undergo cell-autonomous inflammatory activation, possibly due to residual ATP resulting from the specific culture conditions, and/or that factors present *in vivo* that serve to reduce or inhibit inflammatory signals are absent or not sufficiently active in *in vitro* OC cultures. It is not clear that IL-1-dependent signaling is specifically required for this *in vitro* phenomenon, as IL-1R-deficient mice do not exhibit differences in OC size or numbers during *in vitro* RANKL-mediated OC cultures (our unpublished observation). Regardless, our finding that treatment of OC cultures with IL-1β enhances large (>100 μm) TRAP^+^ MNCs numbers (Fig. 7F) suggests that IL-1R-activated signaling promotes multinucleation. It may therefore be important to determine whether there is any relevant cross-talk between IL-1R- and P2X5-mediated signaling mechanisms. In this regard, IL-1R signaling is a major activator of the NF-κB pathway^38^, which has been shown to promote multinucleation^39, 40^. Future work may investigate whether P2X5-dependent multinucleation is linked to NF-κB activation.

We have identified and begun to characterize an ATP-dependent mechanism by which OCs may enhance their activity via inflammasome-triggered IL-1β production. It is possible that this mechanism may utilize other members of the P2X family depending on the specific *in vivo* homeostatic or inflammatory context. In particular, P2X7, which has been shown both to activate the inflammasome pathway^20, 41^, and to regulate ATP-dependent NF-κB activation in OCs^42^, has been studied as a potential target for OC regulation^16, 43^. However, despite promising findings from studies employing chemical inhibitors of P2X7, P2X7-deficient mice exhibit relatively unremarkable bone-related phenotypes both *in vitro* and *in vivo* ^44^. Thus, a clear genetic link between the P2X family and bone has remained elusive. Interestingly, P2X2/P2X5 hetero-oligomers show functional similarity with P2X7 homo-oligomers^45^. Therefore, it is possible that some functional redundancy or hierarchy exists for P2X family groupings expressed in OCs. Also, as alluded to above, it is important to remember that the biology of P2X signaling in OCs may have species-specific features which may be relevant to future studies involving the role of P2X5 in human cells or patient samples. Genotyping of human DNA samples has revealed that a SNP which results in a truncated form of P2X5 is present exclusively in white American, Middle Eastern, and Chinese donors, while samples from African American donors are more polymorphic^23^. Therefore, only people of African descent carry the allele orthologous to the WT allele found in all other examined species, including mice^23^, while most human sub-populations carry only alleles that code for what appears to be a non-functioning P2X5 protein. However, it remains unclear whether expression of truncated human P2X5 exerts a dominant-negative effect when hetero-oligimerizing with other P2X family members, or whether having functional P2X5 correlates with susceptibility to any disease phenotypes or sensitivity to infectious agents in the sub-populations that carry the conserved allele. These allelic variants may correlate to phenotypes that are significant to human health, but making such determinations will first require better understanding of P2X5 function in physiologic contexts.

Our data showing that apyrase treatment affects OC maturation in a manner similar to suramin treatment suggests that ATP can be a functional ligand for relevant OC signaling mechanisms. As we reported (Fig. 7D and E), basal levels of ATP in standard *in vitro* OC cultures are sufficient to drive hyper-multinucleation, but it is not clear whether the same stimuli are relevant in an *in vivo* inflammatory milieu. It remains possible that alternative purinergic ligands are involved in P2X-dependent OC maturation and function. Similarly, with respect to our findings regarding LPS-induced inflammatory bone loss *in vivo*, while we did show that P2X5-dependent effects are required both for increased OC numbers and OC size, together contributing to an increase in bone cavity size, we do not assert that any one P2X-related ligand or stimulus (such as IL-1β) is responsible for these effects. Additional work must be carried out to determine what specific factors regulate P2X-driven inflammatory bone loss *in vivo*.

Overall however, our results not only identify a specific gene target, *P2xr5*, that may be relevant for developing treatments for inflammatory bone loss, but more broadly, highlight a novel strategy for identifying additional genes that enable discernment of normal bone function and pathologic bone loss.

## Methods

### Reagents and antibodies

Suramin (Cat. S2671), ATP (Cat. A6419), TRAP staining (387A-1KT), sodium tartrate (Cat. S4797), pNPP (Cat. N2765), LPS (Cat. L2630) and apyrase (Cat. A6535) were from Sigma-Aldrich (St Louis, MO, USA). Antibodies used in immunoblots to NFATc1 (Cat. sc-7294), c-Src (Cat. Sc-19) and IL-1β (Cat. sc-7884), and for the P2X5 blocking experiment (Cat. sc-15192) were from Santa Cruz Biotechnology (Santa Cruz, CA, USA), to DC-STAMP (Cat. MABF39) was from EMD Millipore (Bedford, MA, USA), to caspase-1 (Cat. AG-20B-0042-c100) was from Adipogen (San Diego, CA, USA), to P2X5 (Cat. ARP35511_P050) was from Aviva systems biology (San Diego, CA, USA). Unless indicated otherwise, all other reagents were from Sigma-Aldrich.

### Mice

*P2rx5* ^−/−^ mice (on a C57BL/6 J background) were generated using *P2rx5* ^−/−^ sperm obtained from the International Mouse Strain Resources (IMSR). In each experiment, homozygous *P2rx5* ^+/+^ and *P2rx5* ^−/−^ mice that were littermates generated from the intercross between heterozygous mice were compared. All mice maintained and used in accordance with guidelines approved by the Institutional Animal Care and Use Committee (IACUC) at University of Pennsylvania.

### Cell sorting and RNA sequencing

Preparation of tetraploid osteoclasts (OC4N) and MGCs (MGC4N), and diploid monocytes (BMM2N) was performed following a method described previously^46^. In brief, BMMs derived from Fucci double transgenic mice (FucciS/G2/M-#474 x FucciG1-#639) were cultured with M-CSF + RANKL (for OCs), IL-3 + IL-4 (for MGCs), or M-CSF only (for BMMs) for 66 hours. Then, cells were harvested and stained with Vybrant Dyecycle violet (Life Technologies) and TO-PRO-3 (Life Technologies), and live mKO2^+^ tetraploid cells or live mKO2^+^ diploid cells were sorted using an Aria (BD Biosciences). For preparation of *STAT6* ^−/−^ diploid MGCs (STAT6^−/−^ MGC2N) and monocytes (STAT6^−/−^ MF2C), BMMs derived from STAT6 knockout mice were cultured with IL-3 + IL-4 or M-CSF for 66 hours, and then cells were stained with Vybrant Dyecycle violet and TO-PRO-3, and live diploid cells were sorted. Purity of sorted cells was confirmed by flow cytometry, and was more than 95%. Total RNAs were isolated from sorted cells using TRIzol LS (Thermo Fisher), and cDNA libraries were prepared using TruSeq RNA Sample Preparation v2 (Illumina). The cDNA libraries were subjected for RNA sequencing by Illumina. Sequencing data was analyzed using htseq-count^47^.

### Micro-computed tomography

Tibial metaphysis were scanned by micro-computed tomography (μCT) using a ScanXmate-A100S. Serial tomographic images were acquired, and three-dimensional microstructural images were reconstructed and used to determine the parameters. Calculations were made for bone morphometric parameters including bone volume over total tissue volume (BV/TV), trabecular thickness (Tb.Th), trabecular number (Th.N), trabecular separation (Tb.Sp), and apparent bone mineral density (BMD) by using TRI/3D-BON software (RATOC System).

### Bone histology and histomorphometry

Fixed tibiae were embedded in paraffin as described previously^48^, and longitudinal sections were stained with H&E for OB parameter analysis or stained with TRAP for OC parameter analysis within the primary and secondary spongiosa.

### Osteoclast (OC) differentiation and tartrate-resistant acid phosphatase (TRAP) staining

To generate primary BMMs, whole BM was extracted from the femurs and tibias of mice and incubated in 10 cm petri dishes in α-MEM medium containing 10% fetal bovine serum and M-CSF (5 ng/ml) for overnight. Non-adherent cells were collected and cultured for 3 days with M-CSF (30 ng/ml). For OC differentiation, BMMs were plated at 1 × 10^4^ per well in 96-well cell culture plates and cultured with M-CSF (30 ng/ml) and RANKL (150 ng/ml) for 3 days, as previously described^49^. Cells were stained using the Acid, Phosphatase, Leukocyte (tartrate-resistant acid phosphatase Kit (387A-1KT, Sigma) following the manufacturer’s instructions.

### Retrovirus preparation and infection

For preparation of retroviral particles, Plat-E packaging cells were transfected with pMX vectors employing Lipofectamin 2000 (Invitrogen). After 3 days, the culture medium containing retroviruses was collected and passed through a 0.22 μm syringe filter. BMMs were infected with retrovirus for 24 h in the presence of 120 ng/ml M-CSF and 8 μg/ml Polybrene (Sigma). Cells were selected in the presence of M-CSF and 1 μg/ml puromycin for 2–3 days prior to use as osteoclast precursors.

### Real-time PCR (qPCR) and immunoblot analysis

For qPCR analysis, total RNA was extracted from cells with the use of the Trizol reagent (Invitrogen), and 1–5 μg of total RNA was reversed transcribed by using random hexamer primers and SuperScript III reverse transcriptase (Invitrogen). cDNA corresponding to 10 ng of total RNA was analyzed by qPCR using an ABI PRISM 7300 real time PCR instrument (Applied Biosystems) and specific Taqman® probes as follows: ACP5(Mm00475698_m1), NFATc1 (Mm00479445_m1), c-fos (Mm00487425_m1), Atp6v0d2 (Mm01222963_m1), DC-STAMP (Mm01168058_m1), OSCAR (Mm00558665_m1), Ctsk (Mm00484036_m1), Car2 (Mm00501572_m1), Itgb3 (Mm00443980_m1), and 18 s (Hs99999901_s1). Amplification parameters were 5 min of denaturation and activation at 95 °C and 50 cycles of 30 s at 95 °C and 1 min at 60 °C. The C_T_ method of relative quantification was used to determine the fold change in expression. For immunoblot analysis, Protein was extracted from osteoclast cultures. Cells were washed three times with ice-cold phosphate-buffered saline (PBS) and lysed in ice-cold radio immunoprecipitation (RIPA) lysis buffer (20 mM Tris-HCl, pH 7.5, 150 mM NaCl, 1% NP-40, 0.5% sodium deoxycholate, 1 mM EDTA, 0.1% SDS, protease and phosphatase inhibitor cocktail (Roche)). Cell lysates were centrifuged to remove debris and determined using the Bradford assay. Equal amount of lysates (2–50 μg of protein) were fractionated by SDS-polyacrylamide gel electrophoresis (SDS-PAGE) on a 4–12% gradient gel and transferred onto a polyvinyldifluoride (PVDF) membrane. Western blot analyses with the indicated antibodies.

### LPS-induced bone destruction and histological analysis

Calvarial bone injections were performed as described previously^50^, with some modifications. A one-time dose of PBS or LPS (12.5 mg/kg) was injected into the subcutaneous tissue over the periosteum of 7-week-old male mouse calvaria at a position directly between the eyes. Mice were sacrificed 5 days after LPS injection and calvarial bone was obtained by surgical dissection, fixed with 4% paraformaldehyde in PBS, pH 7.5, overnight, and decalcified in 0.5 M EDTA, pH 7.1, for 12 h. The calvaria were stained for TRAP and hematoxylin-eosin. To conduct histological analysis, the calvaria were horizontally dissected in the space between the frontal and occipital bone, washed with tap water for 30 min, and dehydrated by serially immersing in 70%, 80%, 90% and 100% ethanol in a processor. After treatment with xylene and molding with paraffin, the molded samples were sectioned at 5 μm with a microtome and slides were subjected to histological analysis by staining TRAP^+^ MNCs, and by measuring the area of TRAP^+^ MNC to determine the size of OCs (μm^2^). For these analyses, 10 TRAP^+^ MNCs were randomly selected on each calvaria slide. To measure bone resorption area, the bone cavity was calculated using image analysis (I-solution®, IMT i-solution, Canada and ImageJ, NIH, U.S.A.).

### Cytokine measurements

Measurements of mouse cytokines were performed using commercially available ELISA kits from BD Biosciences. The range of detection is 8–1000 pg/ml for IL-1β.

### Measurement of intracellular and extracellular ATP

Prior to measurement of ATP release, culture medium was removed, the cell layer washed, and cells incubated with serum-free α-MEM. Samples were collected after 3 h and immediately snap-frozen on dry ice for later ATP quantification. Intracellular ATP and ATP release were measured using a ATP colorimetric/Fluorometric Assay Kit (BioVision) according to the manufacturer’s instructions. The standard curve for ATP was made with a series of dilutions. ATP quantifications were normalized for protein concentration determined by the Bradford assay.

### Statistical analysis

Data are shown as mean ± SD from at least three independent experiments. All experiments were analyzed using one-way ANOVA or 2-tailed paired Student’s *t* test by Prism 6.0 (GraphPad Software). *P* < *0.05* was considered statistically significant.

## Electronic supplementary material

Supplementary Information
