# Supplementary material for: The purinergic receptor P2X5 regulates inflammasome activity and hyper-multinucleation of murine osteoclasts
Source: Sci Rep. 2017 Mar 15;7:196. doi: 10.1038/s41598-017-00139-2 (PMC5427844; doi:10.1038/s41598-017-00139-2)
Supplement: Supplementary file 1 — Supplementary Information [file 41598_2017_139_MOESM1_ESM.doc]

**Supplementary Information**

**The purinergic receptor P2X5 regulates inflammasome activity and hyper multinucleation of murine osteoclasts.**

Hyunsoo Kim, Matthew C Walsh, Noriko Takegahara, Sarah A Middleton, Hong-In Shin, Junhyong Kim, and Yongwon Choi*

*To whom correspondence should be addressed. E-mail: [ychoi3@mail.med.upenn.edu](mailto:ychoi3@mail.med.upenn.edu).

**Figure. S1. Inhibitory effect of anti-P2X5 Ab on *P2rx5^+/+^* and *P2rx5^-/-^* BMMs during osteoclast differentiation.** (**A**) Cells were stained for TRAP and (**B**) measured TRAP activity at OC405nm. TRAP^+^ MNCs were counted by the presence of (**C**) more than 3 nuclei and (**D**) cell size larger than 100 μm in diameter. Scale bar represents 100 μm. ***P* < 0.01 and ****P* < 0.001. Data are means ± SD.

**Figure. S2. Comparative analysis of osteoclast differentiation markers in *P2rx5^+/+^* and *P2rx5^-/-^* OCs.** BMMs from *P2rx5^+/+^* and *P2rx5^-/-^* mice were cultured in the presence of M-CSF and RANKL. At days 0, 1, 2 and 3, total RNA was isolated and subjected to reverse transcription and then analyzed by qPCR. All PCR reactions were carried out in triplicate. Acid phosphatase 5, Tartrate resistant (ACP5), nuclear factor of activated T cells, cytoplasmic 1 (NFATc1), c-fos, ATP6V0d2, DC-STAMP, OSCAR, cathepsin K (Ctsk), carbonic anhydrase 2 (Car2) and Integrin β3 (Itgb3) levels were normalized to 18s RNA. ****P* < 0.001. Data are means ± SD.

**Figure. S3. Intracellular and extracellular ATP levels from osteoclasts.** BMMs from *P2rx5^+/+^* and *P2rx5^-/-^* mice were cultured for 2 days in the presence of M-CSF and RANKL, and the (**A**) intracellular ATP levels and (**B**) extracellular ATP are then measured by described *Methods*. NS, not significant. Data are means ± SD.
